# Highly bulky and stable geometry-constrained iminopyridines: Synthesis, structure and application in Pd-catalyzed Suzuki coupling of aryl chlorides

**DOI:** 10.3762/bjoc.13.24

**Published:** 2017-02-03

**Authors:** Yi Lai, Zhijian Zong, Yujie Tang, Weimin Mo, Nan Sun, Baoxiang Hu, Zhenlu Shen, Liqun Jin, Wen-hua Sun, Xinquan Hu

**Affiliations:** 1 College of Chemical Engineering, Zhejiang University of Technology, Hangzhou 310032, P.R. China; 2 State Key Laboratory for Oxo Synthesis and Selective Oxidation, Lanzhou Institute of Chemical Physics, Chinese Academy of Sciences, Lanzhou 730000, P.R. China; 3 Key laboratory of Engineering Plastics and Beijing National Laboratory for Molecular Science, Institute of Chemistry, Chinese Academy of Sciences, Beijing 100190, P.R. China

**Keywords:** aryl chloride, geometry-constrained, iminopyridyl, palladium, Suzuki

## Abstract

A series of bulky geometry-constrained iminopyridylpalladium chlorides were developed. The steric environment adjacent to the nitrogen atom in the pyridine rings and diimine parts enhanced the thermal stability of the palladium species. Bulkier groups at the imino group stabilized the palladium species and the corresponding palladium chlorides showed high activities in the coupling reaction of aryl chlorides.

## Introduction

Palladium-promoted C–C coupling reactions are recognized as one of the most powerful tools in organic syntheses, pharmaceutical processes and biological modifications [1–4]. The achievement is largely beneficial to organic chemists for their parallel contributions in Pd-catalyzed transformations as well as for discovering new reactions. The improvement of the efficiency of noble metal catalysts is still highly attractive from both the academic and the industrial point of view [5]. Targeting the handling and applicability of palladium catalysts, various species have been developed through the use of diversified ligands [6–12].

Typical ligands that successfully enhanced the reactivity of palladium species towards the oxidative addition of aryl or alkyl halides include phosphine ligands independently developed by, for example, Buchwald [13–15], Hartwig [16–17], Fu [18–20], Kwong [21–23], Tang [24–27] and Lundgren [28], and N-heterocyclic carbene ligands due to the inherent strong sigma-donating property [29]. Alternatively, the palladacycles reported by Herrmann and Beller in the 1990s [30] effectively elongated the lifetime of the active Pd species through employing bidentate or multidentate ligands [31–36]. Meanwhile nitrogen ligands are also widely used for palladium catalysts in coupling reactions with the advantage of convenient synthetic methods, easy functional group modifications and better stability [37–43]. Pyridine, azole and imine-based N(sp^2^) ligands received considerable attention, especially bidentate N,N-ligands. In general, most bidentate N,N(sp^2^) ligands comprise symmetrical frameworks, such as bipyridine [44–46], biimidazole [47–48], and diimine [49–53]. Moreover iminopyridines were attractive for a more straightforward preparation, the condensation of pyridine-2-carboxaldehyde or ketone and the relevant primary amines. By this work a wide variety of different substituents were introduced leading to a diversity with regard to steric and electronic aspects [54–59].

**Figure 1:**
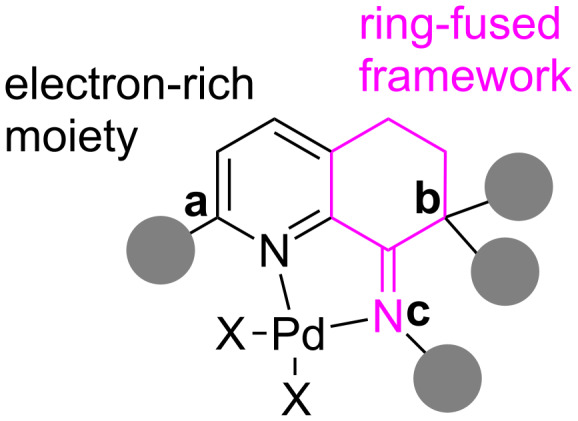
The steric geometry-constrained iminopyridyl–palladium complexes.

Recently, we have developed a series of geometry-constrained iminopyridyl compounds [60–64] and their corresponding palladium complexes showed a good catalytic efficiency for both Suzuki and Heck cross-coupling reactions. The favorable effect originated from the ring-fused framework being able to establish a strained environment for better stability. Efficient couplings were achieved with aryl bromides or iodides as substrates, however, it is still not favored for the cheaper and more widely available aryl chloride substrates. For C–Cl bond activation, the major efforts have focused on using extraordinarily electron-rich ligands to promote the oxidative addition. We assumed that the coupling of aryl chlorides could also be furnished under high temperature, if the palladium catalyst is stable enough. The formation of palladium black was observed in reactions of aryl chlorides using the reported palladium catalyst system, which inspired us to improve the thermal stability of the palladium complex. The steric environment adjacent to the nitrogen in pyridine rings and diimine parts enhanced the thermal stability of metal species, albeit under harsh conditions [65–68]. Therefore, Figure 1 schematically illustrates various substituents of iminopyridines constraining the geometrical influence around the palladium center. In this report, the modifications using various substituents with different electronic and steric factors have been explored, and fortunately thermally stable palladium chlorides are obtained demonstrating high activities toward the coupling reaction of aryl chlorides.

## Results and Discussion

According to Figure 1, the geometry-constrained iminopyridyl skeleton could be modified at three sites: *C*^a^, *C*^b^ and *N*^c^. Both the derivation of the *C*^a^ position and adjusting the imino part are expected to protect the active palladium center from decomposition during the catalytic cycle. Nevertheless, multiple substitutions at *C*^b^ probably prohibit any tautomerization and result to increase the stability of iminopyridines and their palladium catalysts.

**Scheme 1:**
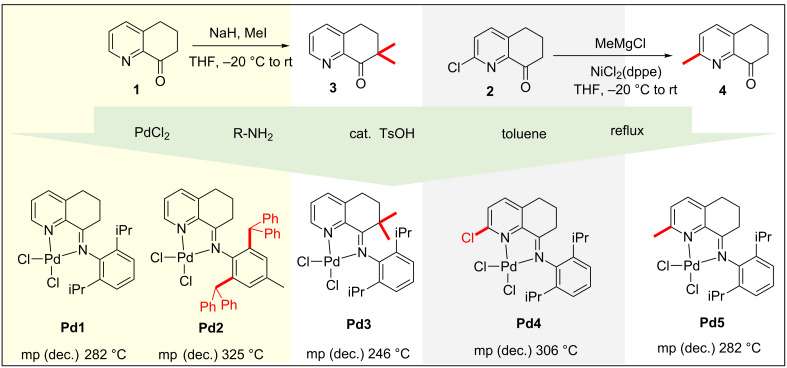
Preparation of the bulky iminopyridyl–palladium complexes.

6,7-Dihydroquinolin-8(5*H*)-one (**1**) and 2-chloro-6,7-dihydroquinolin-8(5*H*)-one (**2**) could be prepared in large amounts following the previously reported synthetic procedures [60,62,69–71]. Geminal dimethylation of **1** with NaH and MeI in THF afforded 7,7-dimethyl-6,7-dihydroquinolin-8(5*H*)-one (**3**) easily in good yield. Meanwhile, the Ni-catalyzed Kumada coupling of **2** with methylmagnesium chloride produced 2-methyl-6,7-dihydroquinolin-8(5*H*)-one (**4**) efficiently with quantitative yield. With compounds **1**, **2**, **3** and **4** in hand, further condensations with different arylamines and direct coordination to PdCl_2_ in one pot smoothly in the presence of TsOH. For this transformation, we found that vigorous stirring during the reaction was very important, which increased the yields from medium to quantitative results [72]. Following a similar procedure as described in the Experimental section, complexes **Pd1** to **Pd5** were synthesized and could be isolated by simple work-up (Scheme 1). All of these complexes have been fully characterized and confirmed by NMR and HRMS, and the analyses are in good agreement with that of the previously reported palladium complexes [72]. All of these complexes could be stored open to air without any decomposition for several months except **Pd4**. Their melting points [>240 °C] indicated their high thermostability, especially **Pd2** with a melting point up to 325 °C.

With these sterically hindered iminopyridine–palladium complexes **Pd1** to **Pd5** in hand, we firstly investigated their catalytic activity directly in Suzuki cross-coupling reactions with chlorobenzene as the electrophile. The reactions were performed under the previously reported conditions without any change except at the higher reaction temperature. As shown in Table 1, using complex **Pd1**, which has exhibited an impressive efficiency in Pd-catalyzed Suzuki coupling of aryl bromides, only delivered 18% conversion of chlorobenzene. The modification of the *C*^b^ position by importing two methyl groups of **Pd1** to **Pd3** did not give any improvement while with much lower conversion. The efforts changing the steric and electronic effect from the *ortho*-position of pyridyl (**Pd4** and **Pd5**) also failed to promote C–Cl bond activation. To our delight, complex **Pd2**, with a bulkier version of dipp (2,6-diisopropylphenyl) in the imino motif, displayed significant enhance with the conversion up to 44%. The results in Table 1 and Figure 1 matched well with our proposal that improving the stability of palladium species favored C–Cl bond activation under higher temperature.

**Table 1:** Pd-catalyzed coupling of chlorobenzene and 4-tolylboronic acid using **Pd1**–**Pd5** as the catalyst.^a^

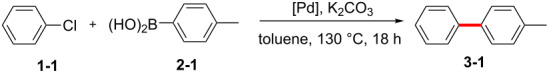					

Cat.	**Pd1**	**Pd2**	**Pd3**	**Pd4**	**Pd5**

Conv.(%)^b^	18	44	5	4	18

^a^Reaction conditions: chlorobenzene (1.0 mmol), 4-methylphenylboronic acid (1.2 mmol), toluene (3.0 mL), Pd catalyst (1 mol %), 18 h, purged with N_2_ for 2 min. ^b^Conversions were determined by GC.

**Figure 2:**
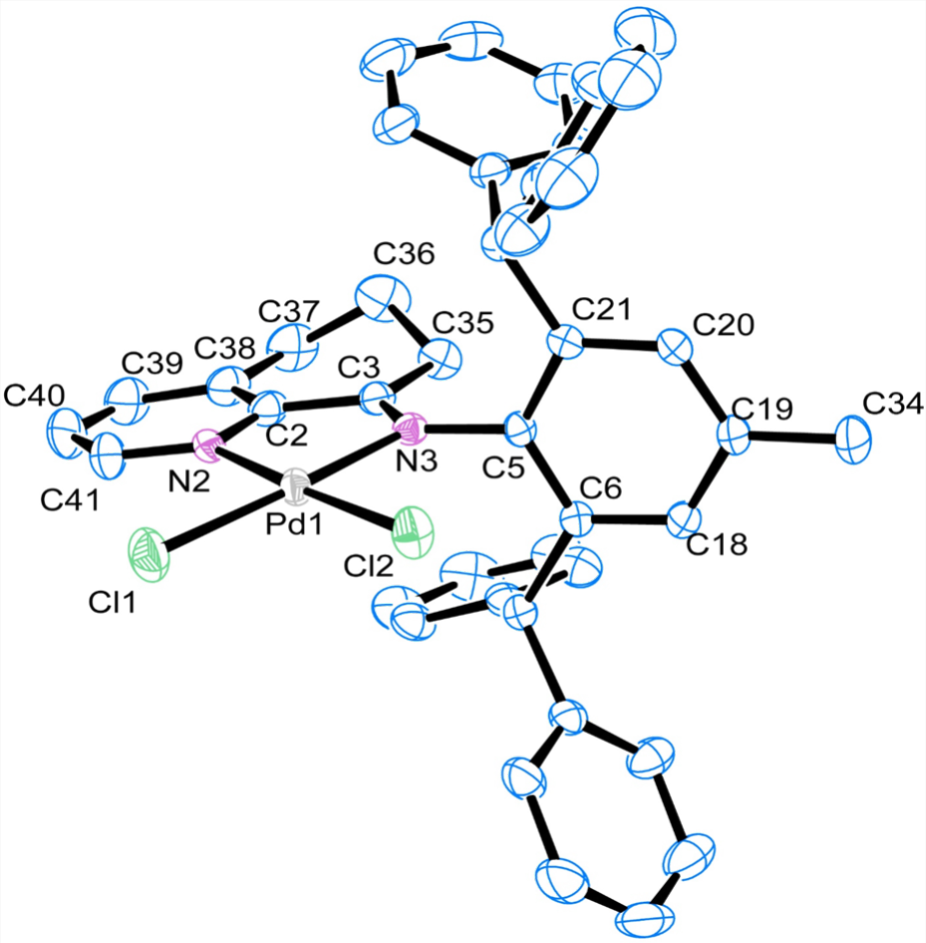
ORTEP drawing of **Pd2** with thermal ellipsoids at 30% probability level. Hydrogen atoms and the solvent CH_2_Cl_2_ have been omitted for clarity.

Single crystals of complex **Pd2** were obtained by slow diffusion of diethyl ether to a saturated dichloromethane solution and the molecular structure in solid state was determined by X-ray diffraction [73]. Presented in Figure 2, the molecular structure and the predicted conformation was unambiguously confirmed. The same as before, the complexes also exhibit square-planar coordination geometry around the palladium centre with a slight distortion. Selected bond lengths and angles listed in Table 2 suggest that both the imino double bond N3–C3 (1.304 Å) and Pd–N3 (2.037 Å) were slightly longer than that of our previous reported iminopyridyl–palladium complexes (1.27–1.30 Å, 2.02–2.03 Å, respectively) [57]. The structure clearly showed a flexible steric bulky effect from the replacement of two methyl groups of iPr by phenyl rings and the palladium center could be protected very well.

**Table 2:** Selected bond lengths [Å] and angles [°] for complexes **Pd2**.

Bond lengths (Å)		Bond angles (°)	

Pd(1)–N(2)	2.036 (3)	N(3)–Pd(1)–N(2)	80.45(9)
Pd(1)–N(3)	2.038(2)	N(3)–Pd(1)–Cl(1)	174.33(7)
Pd(1)–Cl(2)	2.2772(10)	N(3)–Pd(1)–Cl(2)	94.80(6)
Pd(1)–Cl(1)	2.2938(9)	N(2)–Pd(1)–Cl(2)	175.22(7)
N(2)–C(2)	1.373(4)	N(2)–Pd(1)–Cl(2)	93.99(7)
N(2)–C(41)	1.327(4)	Cl(2)–Pd(1)–Cl(1)	90.75(3)
N(3)–C(3)	1.304(3)		
N(3)–C(5)	1.437(3)		

With **Pd2** as the catalyst, we then put our efforts on simply optimizing the reaction conditions for the coupling between chlorobenzene and 4-methylphenylboronic acid. We noticed that water could promote the conversion of chlorobenzene to 62% which might be due to the better solubility of the base in toluene/H_2_O (2.5:1) mixed solvents (Table 3, entry 1). Bases have been proven to play an important role in Suzuki coupling reactions [74]. Then, in mixed solvents, various bases were examined (Table 3, entries 1–10). Compared with K_2_CO_3_, the other tested bases did not show any advantage. Increasing the amount of base to 2.5 equivalents or elevating the temperature to 140 °C could be an advantage for the reaction. Without any other screening, we finally determined the reaction conditions as following for the next substrates scope test: **Pd2** (1 mol %), toluene/H_2_O (5:1), 24 h, 140 °C.

**Table 3:** The optimization of conditions for Pd-catalyzed coupling reactions between chlorobenzene and phenylboronic acid ^a^.

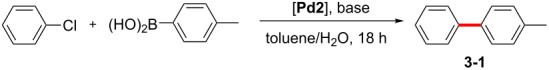			

Entry	Base (equiv)	Temperature (°C)	Conversion.(%)^b^

1	K_2_CO_3_ (2.0)	130	62
2	Cs_2_CO_3_ (2.0)	130	31
3	*t*-BuOK (2.0)	130	trace
4	NaOH (2.0)	130	16
5	DIPEA (2.0)	130	NR
6	KOAc (2.0)	130	8
7	Na_2_CO_3_ (2.0)	130	22
8	NaF (2.0)	130	NR
9	NaOAc (2.0)	130	NR
10	EtONa (2.0)	130	30
11	K_2_CO_3_ (2.2)	130	69
12	K_2_CO_3_ (2.5)	130	72
13	K_2_CO_3_ (2.2)	100	52
14	K_2_CO_3_ (2.2)	120	54
15	K_2_CO_3_ (2.2)	140	77
16	K_2_CO_3_ (2.2)	150	74

^a^Reaction conditions: chlorobenzene (1.0 mmol), 4-methylphenylboronic acid (1.2 mmol), toluene/H_2_O (2.5/0.5, v/v), **Pd2** (1 mol %), 18 h, purged with N_2_ for 2 min. ^b^Conversions were determined by GC. DIPEA = diisopropylethylamine.

With the optimized reaction conditions, a standard reaction in a 5 mmol scale between chlorobenzene and phenylboronic acid was carried out and 64% isolated yield was obtained (Table 4, entry 1). Subsequently, different aryl chlorides and arylboronic acids were tested. The results were listed in Table 4. Aryl chlorides with an electron-withdrawing functional group, such as nitro (Table 4, entry 2), carbonyl (Table 4, entries 4–6), nitrile and CF_3_ (Table 4, entries 6 and 7) could be coupled with 4-methylphenylboric acid in good to excellent yields. Aryl chlorides with an electron-donating functional group (Table 4, entries 9 and 10) and steric hindered 2-chlorotoluene (Table 4, entry 11), which are usually regarded as reluctant substrates due to the sluggish oxidative addition of Pd(0) to aryl chlorides, proceeded successfully with moderate yields. Heterocyclic aryl chloride, for example, 2-chloropyridine, was also well tolerated and 84% isolated yields was obtained (Table 4, entries 13). Different arylboronic acids were also examined to react with 4-chloroacetophenone in this system. No matter what kind of boronic acids were used: electron-deficient (Table 4, entry 14), electron-rich (Table 4, entries 15 and 16) or sterically hindered (Table 4, entry 17) arylboronic acids, high conversions and excellent isolated yields were achieved.

**Table 4:** Pd-catalyzed coupling between various aryl chlorides and arylboronic acids.^a^

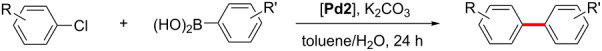			

Entry	ArCl	ArB(OH)_2_	Conv/Yield (%)^b^

1	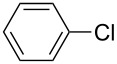 **1-1**	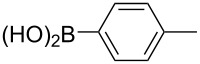 **2-1**	**3-1** 71/64
2	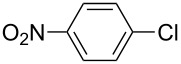 **1-2**	**2-1**	**3-2** 64/58
3	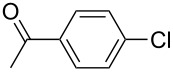 **1-3**	**2-1**	**3-3** 99/99
4	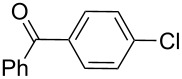 **1-4**	**2-1**	**3-4** 86/85
5	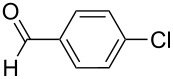 **1-5**	**2-1**	**3-5** 82/69
6	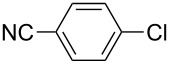 **1-6**	**2-1**	**3-6** 94/90
7	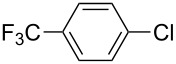 **1-7**	**2-1**	**3-7** 93/86
8	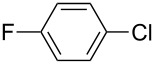 **1-8**	**2-1**	**3-8** 80/75
9	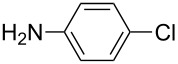 **1-9**	**2-1**	**3-9** 60/49
10	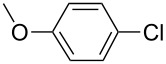 **1-10**	**2-1**	**3-10** 59/51
11	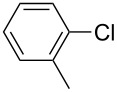 **1-11**	**2-1**	**3-11** 64/57
12	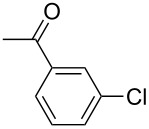 **1-12**	**2-1**	**3-12** 91/91
13	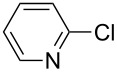 **1-13**	**2-1**	**3-13** 92/84
14	**1-3**	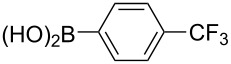 **2-2**	**3-14** 99/95
15	**1-3**	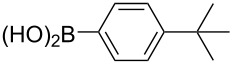 **2-3**	**3-15** 99/99
16	**1-3**	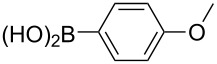 **2-4**	**3-16** 96/89
17	**1-3**	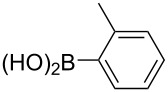 **2-5**	**3-17** 95/93

^a^Reaction conditions: aryl chlorides (5.0 mmol), arylboronic acid (6.0 mmol), K_2_CO_3_ (11.0 mmol), toluene/H_2_O (10 mL/2 mL), **Pd2** (1 mol %), 140 °C, 24 h, purged with N_2_ for 2 min. ^b^Conversions were determined by GC and yields were isolated results.

## Conclusion

In summary, a series of geometrically-constrained iminopyridine–palladium complexes were prepared with various substituents. They promoted efficiently the Suzuki cross coupling of aryl chlorides. To result good catalytic activities the importance of bulkier groups at the imino moiety for the stabilization of the palladium species was sown. A variety of aryl chlorides and arylboronic acids were successfully coupled in high yields and selectivity.

## Experimental

### General procedures

All reactions were carried out under air atmosphere unless otherwise noted. All ^1^H and ^13^C NMR spectra were recorded on a Bruker AVANCE III 500 MHz spectrometer in deuterated solvents with tetramethylsilane (TMS) as internal standard. NMR multiplicities are abbreviated as follows: s = singlet, d = doublet, t = triplet, q = quartet, sept = septet, m = multiplet, br = broad signal. Chemical shifts are given in ppm and are referenced to SiMe_4_ (^1^H, ^13^C) and all spectra were obtained at 25 °C in the solvent indicated. Coupling constants *J* are given in Hz. GC analyses were performed on Agilent 6890 instrument with FID detector using an HP-5 capillary column (30 m × 0.32 mm (i.d.), 0.25 μ). High-resolution mass spectra were recorded in the EI mode on Agilent 6210 TOF mass spectrometry. Flash column chromatography was performed on neutral SiO_2_ (200–300 mesh) with ethyl acetate/petroleum as eluent. Melting points were determined on BÜCHI M-565 apparatus. X-ray crystallography were performed on a Bruker Smart Apex CCD area detector diffractometer using graphite-monochromated Mo Kα radiation (λ = 0.71073 Å). Details of the X-ray structure determinations and refinements are provided in Table 5. According to the synthetic procedures in the literature, the organic compounds **1**, **2**, **3**, and **4** were prepared in good yield [69,71,75].

**Table 5:** Crystal data and structure refinement for **Pd2**.

Identification code	**Pd2**	Identification code	**Pd2**

empirical formula	C_42_H_34_N_2_Cl_2_Pd·CH_2_Cl_2_	ρcalcg/cm^3^	1.4237
formula weight	829.00	μ/mm^−1^	0.789
crystal system	orthorhombic	F(000)	3373.8
temperature/K	173	radiation	Mo Kα (λ = 0.71073)
space group	Pbca	2θ range for data collection/^o^	3.58 to 56.6
a/Å	18.996(3)	index ranges	−24 ≤ h ≤25, −26 ≤ k ≤ 26, −27 ≤ l ≤ 23
b/Å	19.832(4)	reflections collected	66946
c/Å	20.531(4)	independent reflections	9585 [Rint = 0.0438, Rsigma = 0.0275]
α/^o^	90	data/restraints/parameters	9585/0/470
β/^o^	90	goodness-of-fit on F^2^	1.123
γ/^o^	90	final R indexes [I>=2σ (I)]	R1 = 0.0412, wR2 = 0.1026
volume/Å^3^	7735(2)	final R indexes [all data]	R1 = 0.0714, wR2 = 0.1295
Z	8	largest diff. peak/hole/e Å^−3^	1.09/−0.68

#### Synthesis of **Pd2**

Toluene (100 mL) was added to a mixture of 6,7-dihydroquinolin-8(*5H*)-one (1.57 g, 11 mmol), 2,6-dibenzhydryl-4-methylaniline (5.27 g, 12 mmol), PdCl_2_ (1.77 g, 10 mmol) and TsOH·H_2_O (0.95 g, 5 mmol). After being degassed by N_2_ for 2 min, the reaction mixture was sealed and stirred at 130 °C for 36 hours. Then the tube was allowed to cool to room temperature. After filtration and washing with toluene, the resulted residue was purified by column chromatography with dichloromethane as the eluent. The complex **Pd2** was obtained as yellow solid (7.1 g, yield 95%). Mp (dec.) 325 °C. ^1^H NMR (CDCl_3_, 500 MHz) δ 9.42 (t, *J* = 3.4 Hz, 1H), 7.70 (d, *J* = 3.4 Hz, 2H), 7.63 (m, 16H), 7.30 (d, *J* = 7.4Hz, 4H), 6.72 (s, 2H), 6.22 (s, 2H), 2.14 (s, 5H), 0.48 (t, *J* = 6.5 Hz, 2H), 0.17 (m, 2H); ^13^C NMR (CDCl_3_, 125 MHz) δ 182.1, 151.9, 149.9, 143.2, 142.4, 141.4, 140.3, 137.3, 137.0, 130.1, 129.9, 129.3, 129.0, 128.2, 127.0, 126.5, 53.6, 52.3, 31.6, 31.0, 27.6, 21.7, 19.8; HRMS (ESI+) *m*/*z*: [M – Cl + CH_3_CN]^+^ calcd for C_42_H_36_ClN_2_Pd·CH_3_CN, 750.1874; found, 750.1897.

#### Synthesis of **Pd3**

Using xylene as the solvent, and following a similar procedure to that described for **Pd2** at 160 °C, resulted **Pd3** with 29% yield as yellow powder. Mp 246 °C; ^1^H NMR (DMSO-*d*_6_, 500 MHz) δ 9.10 (d, *J* = 5.2 Hz, 1H), 8.22 (d, *J* = 7.8 Hz, 1H), 7.87 (m, 1H), 7.26 (m, 1H), 7.14 (d, *J* = 7.7 Hz, 2H), 3.06 (m, 4H), 1.86 (t, *J* = 6.1 Hz, 2H), 1.45 (d, *J* = 6.7 Hz, 6H), 1.26 (d, *J* = 6.7 Hz, 6H), 0.89 (s, 6H); ^13^C NMR (DMSO-*d*_6_, 125 MHz) δ 181.7, 153.4, 150.2, 142.5, 140.7, 140.5, 139.6, 128.4, 128.3, 123.4, 42.5, 37.7, 29.3, 29.0, 27.4, 25.7, 24.8, 24.5, 23.7; HRMS (ESI+) *m*/*z*: [M – Cl + CH_3_CN]^+^ calcd for C_23_H_30_ClN_2_Pd·CH_3_CN, 518.1392; found, 518.1378.

#### Synthesis of **Pd4:**

Toluene (4.0 mL) was added to a mixture of 2-chloro-6,7-dihydroquinolin-8(5*H*)-one (181 mg, 1 mmol), 2,6-dibenzhydryl-4-methylaniline (468 mg, 1.1 mmol), and TsOH·H_2_O (57 mg, 0.3 mmol). After degassed by N_2_ for 2 min, the reaction mixture was sealed and stirred at 130 °C for 24 hours. Then the tube was allowed to cool to room temperature and PdCl_2_ (160 mg, 0.9 mmol) was added. After stirring at 50 °C for additional 24 hours, filtration and washing with toluene, the resulted residue was purified by column chromatography with dichloromethane as the eluent. The complex **Pd4** was obtained as yellow solid (380 mg, yield 82%). Mp (dec.) 306 °C. **Pd4** decomposes slowly when exposed open to air. ^1^H NMR (CDCl_3_, 500 MHz) δ 7.90 (d, *J* = 8.1 Hz, 1H), 7.69 (d, *J* = 8.1 Hz, 1H), 7.34 (d, *J* = 7.6 Hz, 1H), 7.20 (d, *J* = 7.6 Hz, 2H), 3.13 (m, 2H), 3.03 (m, 2H), 2.44 (m, 2H), 1.93 (m, 2H), 1.44 (d, *J* = 6.7 Hz, 6H), 1.15 (d, *J* = 6.7 Hz, 6H); HRMS (ESI+) *m*/*z*: [M − Cl + CH_3_CN]^+^ calcd for C_21_H_25_Cl_2_N_2_Pd·CH_3_CN, 524.0684; found, 524.0679.

#### Synthesis of **Pd5:**

In a similar procedure to that described for **Pd2** with 81% yield as yellow powder. Mp (dec.) 282 °C; ^1^H NMR (DMSO-*d*_6_, 500 MHz) δ 8.05 (d, *J* = 7.8 Hz, 1H), 7.70 (d, *J* = 7.8 Hz, 1H), 7.30 (t, *J* = 8.1 Hz, 1H), 7.19 (t, *J* = 8.1 Hz, 2H), 3.11 (m, 2H), 2.97 (d, *J* = 8.3 Hz, 5H), 2.40 (t, *J* = 6.0 Hz, 2H), 1.82 (t, *J* = 6.2 Hz, 2H), 1.33 (d, *J* = 6.9 Hz, 5H), 1.10 (d, *J* = 6.8 Hz, 6H); ^13^C NMR (DMSO-*d*_6_, 125 MHz) δ 163.8, 151.6, 141.2, 140.9, 140.2, 139.8, 132.1, 127.9, 123.4, 31.9, 27.9, 27.7, 27.2, 23.6, 23.5, 21.0; HRMS (ESI+) *m*/*z*: [M – Cl + CH_3_CN]^+^ calcd for C_22_H_28_ClN_2_Pd·CH_3_CN, 504.1235; found, 504.1231.

### General procedure for Pd-catalyzed Suzuki cross-coupling reactions

To a Young tube, aryl chlorides (5.0 mmol), K_2_CO_3_ (1.5 g, 11 mmol), arylboric acid (6 mmol), complex **Pd2** (37.5 mg, 1 mol %), toluene (10 mL) and H_2_O (2 mL) were added. The mixture was degassed for 2 min. Then, the sealed Young tube was set into the pre-heated 140 °C oil bath. After stirring for 24 hours, the Young tube was allowed to cool to room temperature. After filtration and extraction with toluene (50 mL), the resulted solution was concentrated under vacuum and the desired biaryl was isolated by column chromatography.

## Supporting Information

File 1 NMR spectra of palladium complexes and products.
