# Supplementary material for: Highly bulky and stable geometry-constrained iminopyridines: Synthesis, structure and application in Pd-catalyzed Suzuki coupling of aryl chlorides
Source: Beilstein J Org Chem. 2017 Feb 3;13:213–21. doi: 10.3762/bjoc.13.24 (PMC5301655; doi:10.3762/bjoc.13.24)
Supplement: File 1 — NMR spectra of palladium complexes and products. [file Beilstein_J_Org_Chem-13-213-s001.pdf]

Supporting Information

for

Highly bulky and stable geometry-constrained iminopyridines: Synthesis, structure and application in Pd-catalyzed Suzuki coupling of aryl chlorides

Yi Lai[1], Zhijian Zong[1], Yujie Tang[1], Weimin Mo[1], Nan Sun[1], Baoxiang Hu[1], Zhenlu Shen[1], Liqun Jin*[1,2], Wen-hua Sun*[3] and Xinquan Hu*[1,2]

Address: [1]College of Chemical Engineering, Zhejiang University of Technology, Hangzhou 310032, P.R. China, [2]State Key Laboratory for Oxo Synthesis and Selective Oxidation Lanzhou Institute of Chemical Physics Chinese Academy of Sciences, Lanzhou 730000, P.R. China and [3]Key laboratory of Engineering Plastics and Beijing National Laboratory for Molecular Science, Institute of Chemistry, Chinese Academy of Sciences, Beijing 100190, P.R. China

Email: Liqun Jin* - liqunjin@zjut.edu.cn; Wen-hua Sun* - whsun@iccas.ac.cn; Xinquan Hu* - xinquan@zjut.edu.cn.

*Corresponding author

NMR spectra of palladium complexes and products

1. NMR spectrum of iminopyridine–palladium complexes

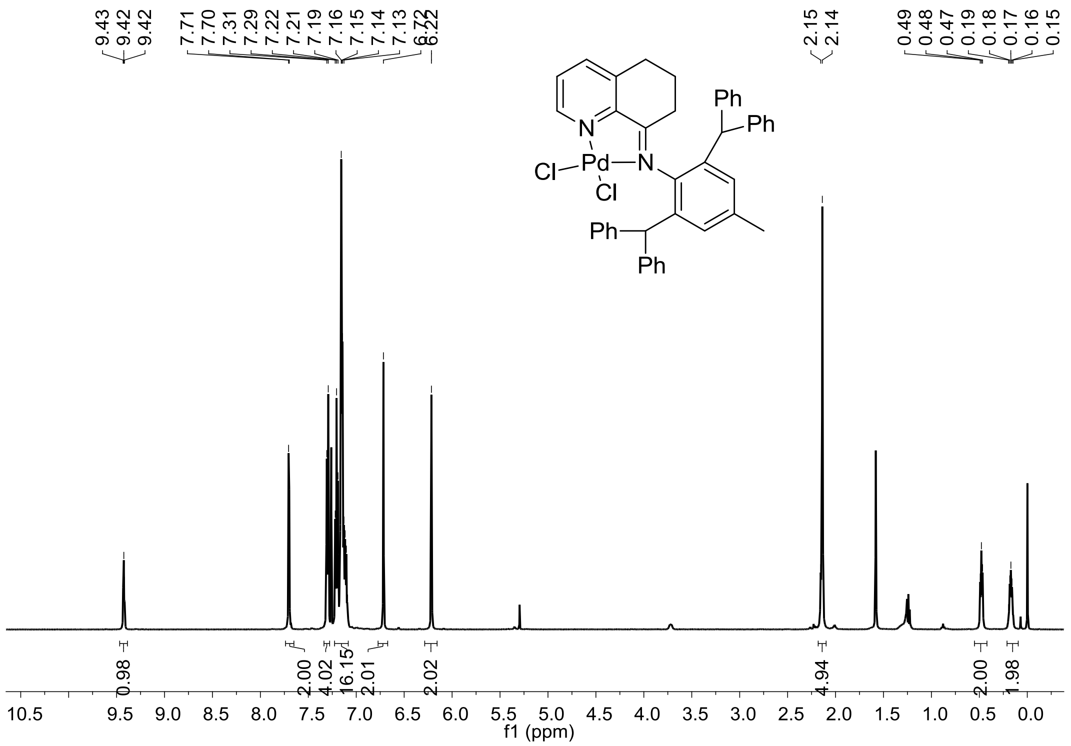

Figure S1 ^{1}H NMR of complex **Pd2** in $CDCl_3$

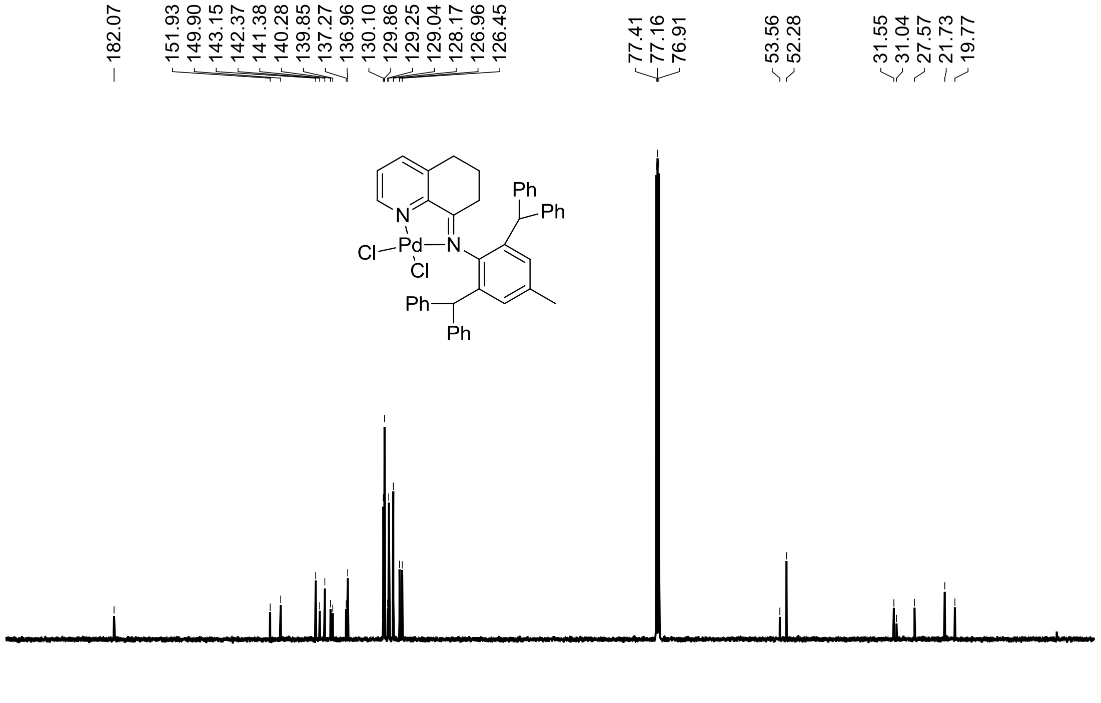

Figure S2 ^{13}C NMR of complex **Pd2** in DMSO-d_6.

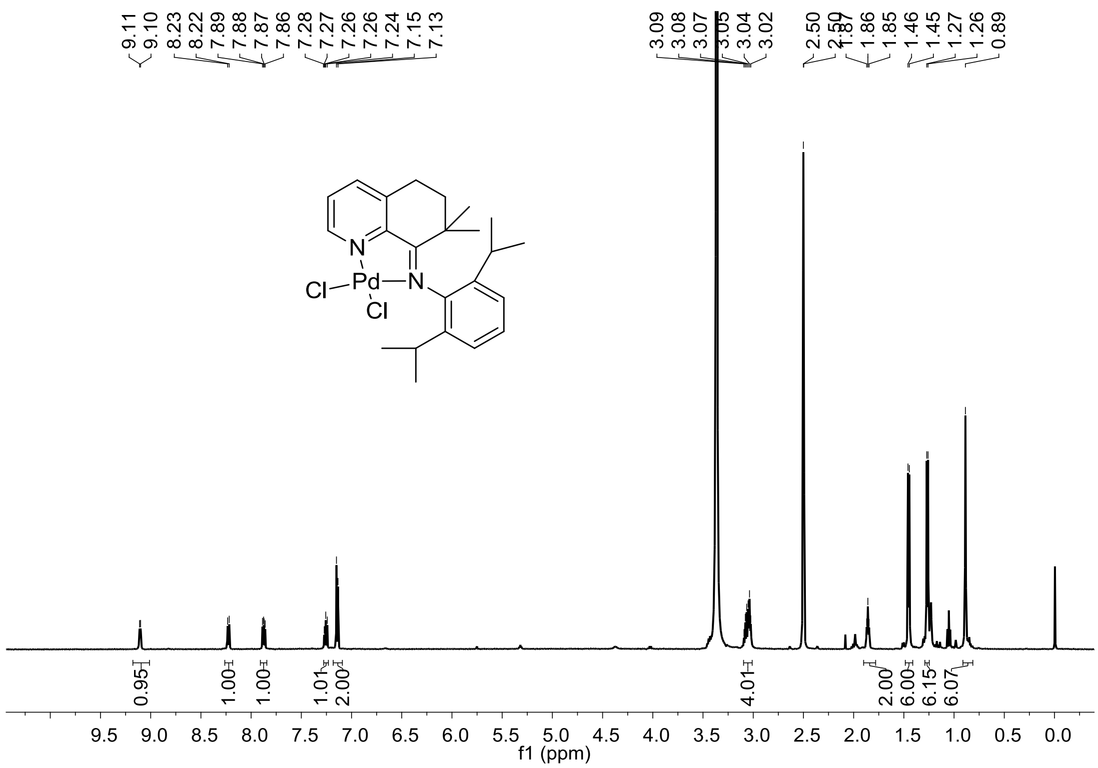

Figure S3 ^{1}H NMR of complex **Pd3** in DMSO-d_6

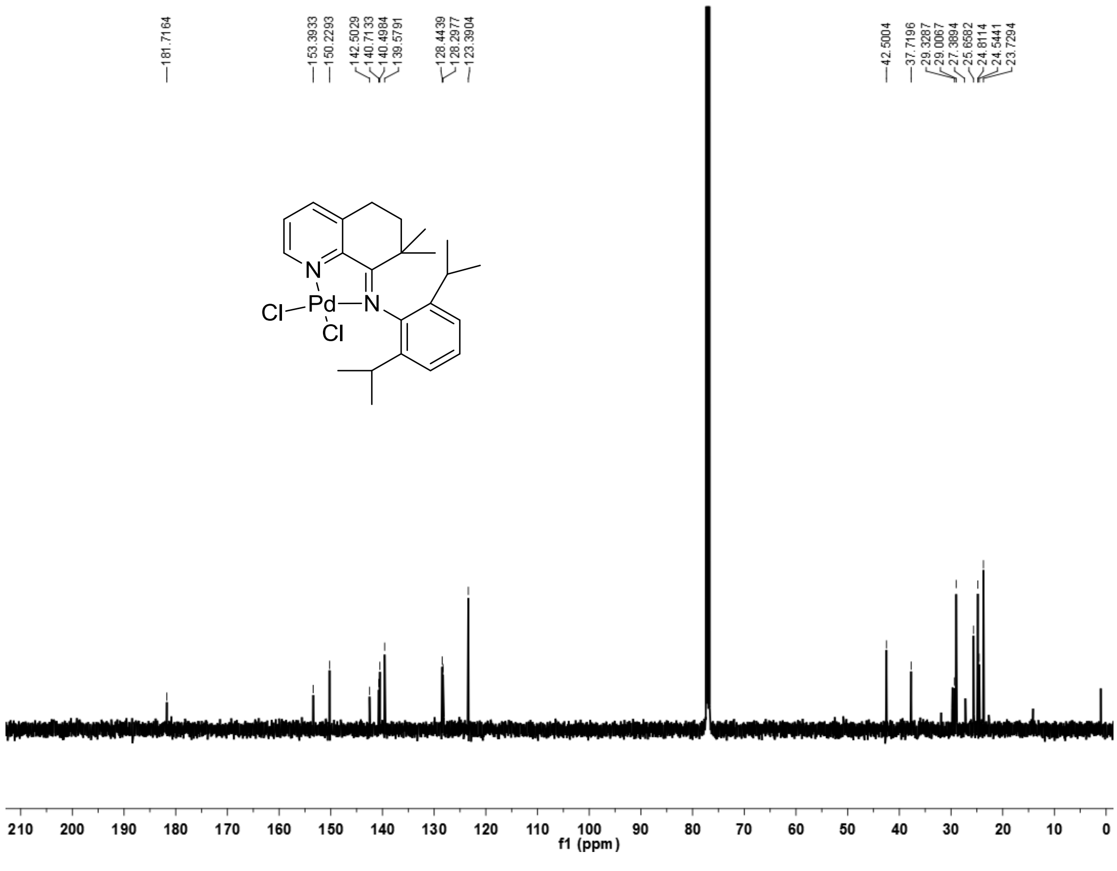

Figure S4 ^{13}C NMR of complex **Pd3** in DMSO-d_6.

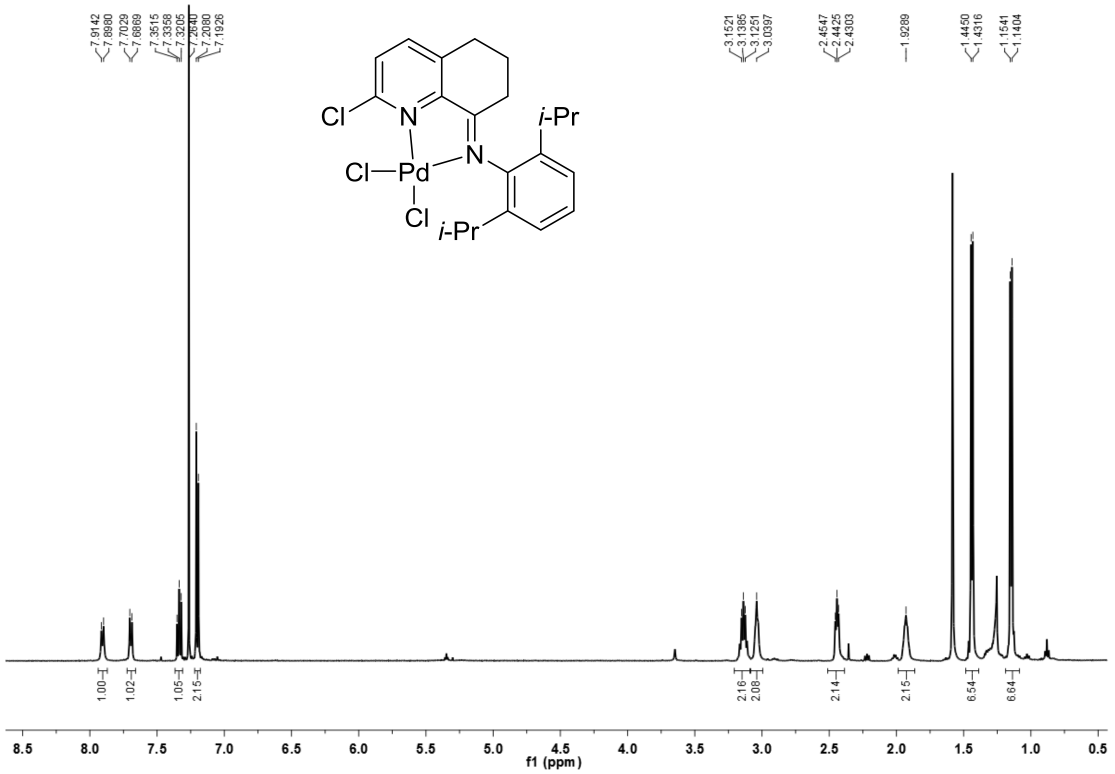

Figure S5 ^{1}H NMR of complex **Pd4** in $CDCl_3$.

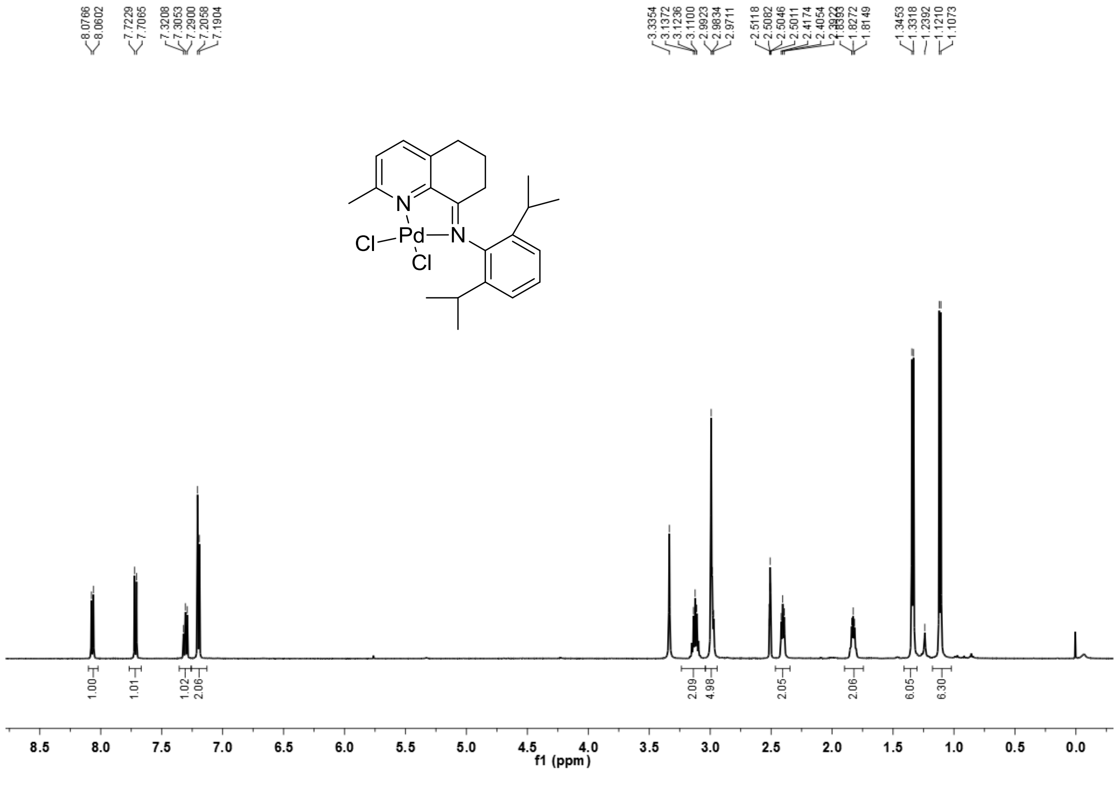

Figure S6 1H NMR of complex **Pd5** in DMSO-d_6.

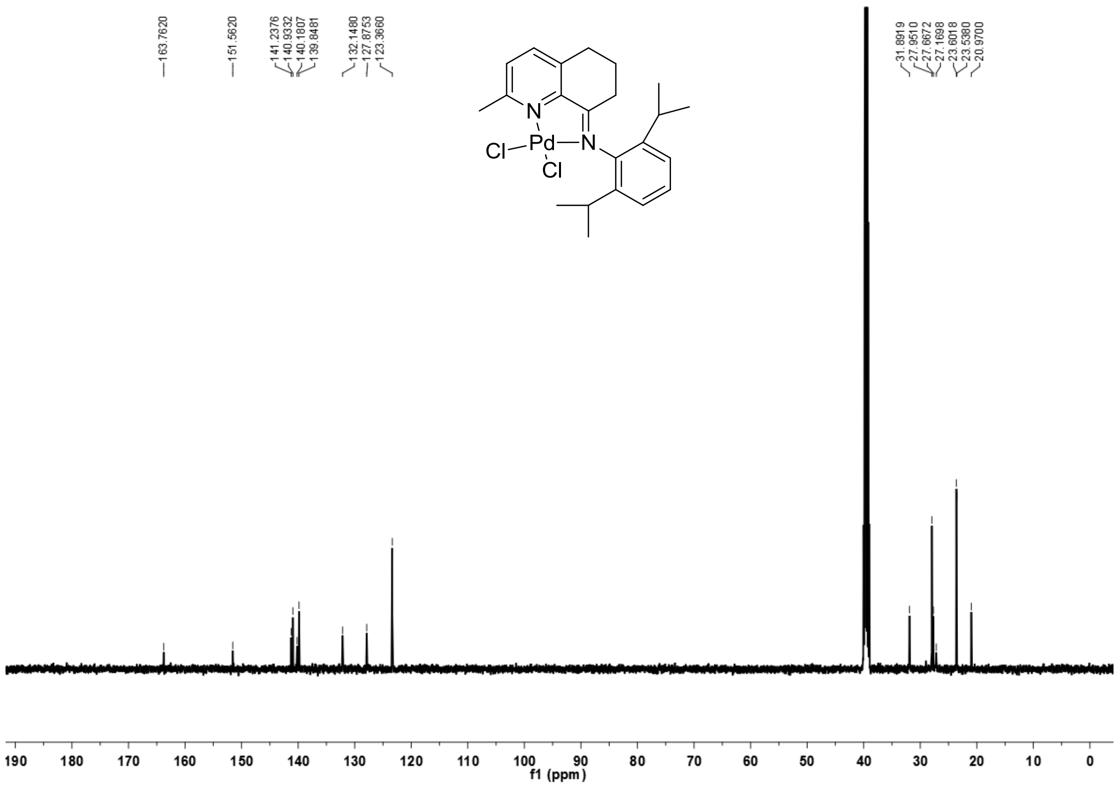

Figure S7 ^{13}C NMR of complex **Pd5** in DMSO-d_6.

2. NMR spectrum of biaryl compounds

4-Methyl-biphenyl (**3-1**)[1]

^{1}H NMR ($CDCl_3$, 500 MHz): δ 7.60 (d, *J* = 8.2Hz, 2H), 7.52 (d, *J* = 8.2 Hz, 2H), 7.45 (t, *J* = 7.4 Hz, 2H), 7.35 (t, *J* = 7.4 Hz, 1H), 7.27 (d, *J* = 7.4 Hz, 2H), 2.42 (s, 3H).

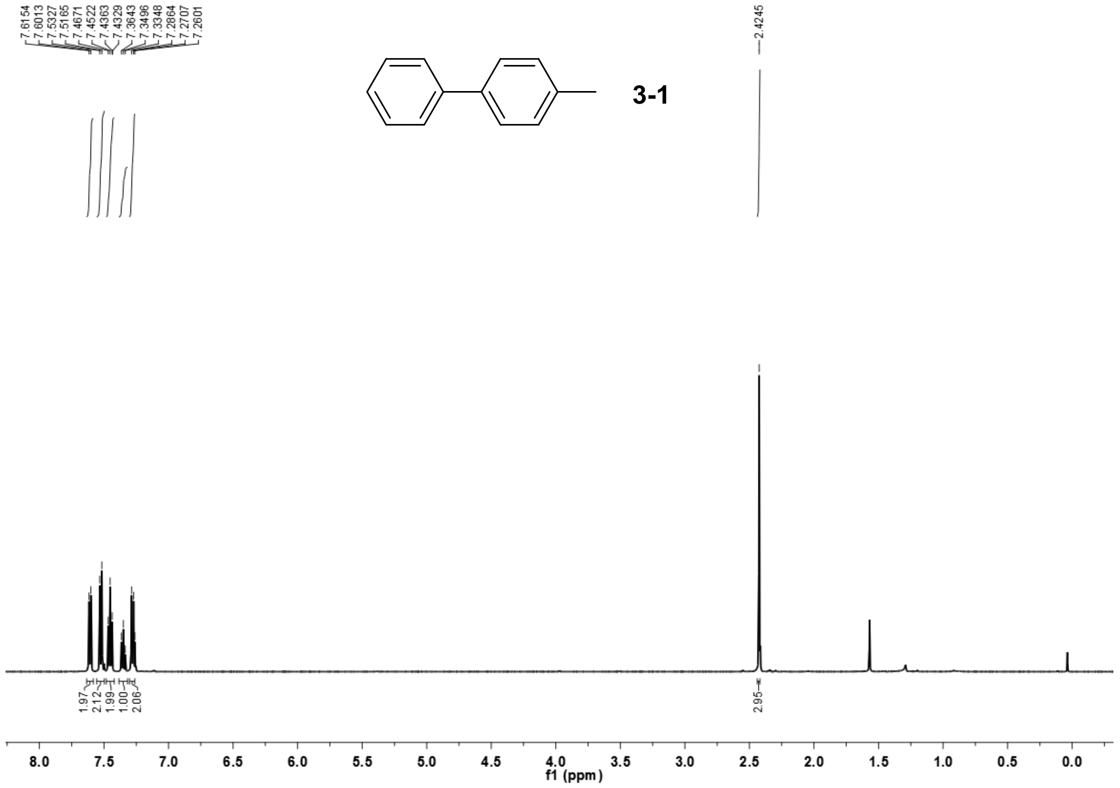

4-Methyl-4'-nitro-biphenyl (**3-2**) [2]

^{1}H NMR ($CDCl_3$, 500 MHz): δ 8.28 (d, *J* = 9.0 Hz, 2H), 7.72 (d, *J* = 9.0 Hz, 2H), 7.53 (d, *J* = 8.1 Hz, 2H), 7.30 (d, *J* = 8.1 Hz, 2H), 2.42 (s, 3H).

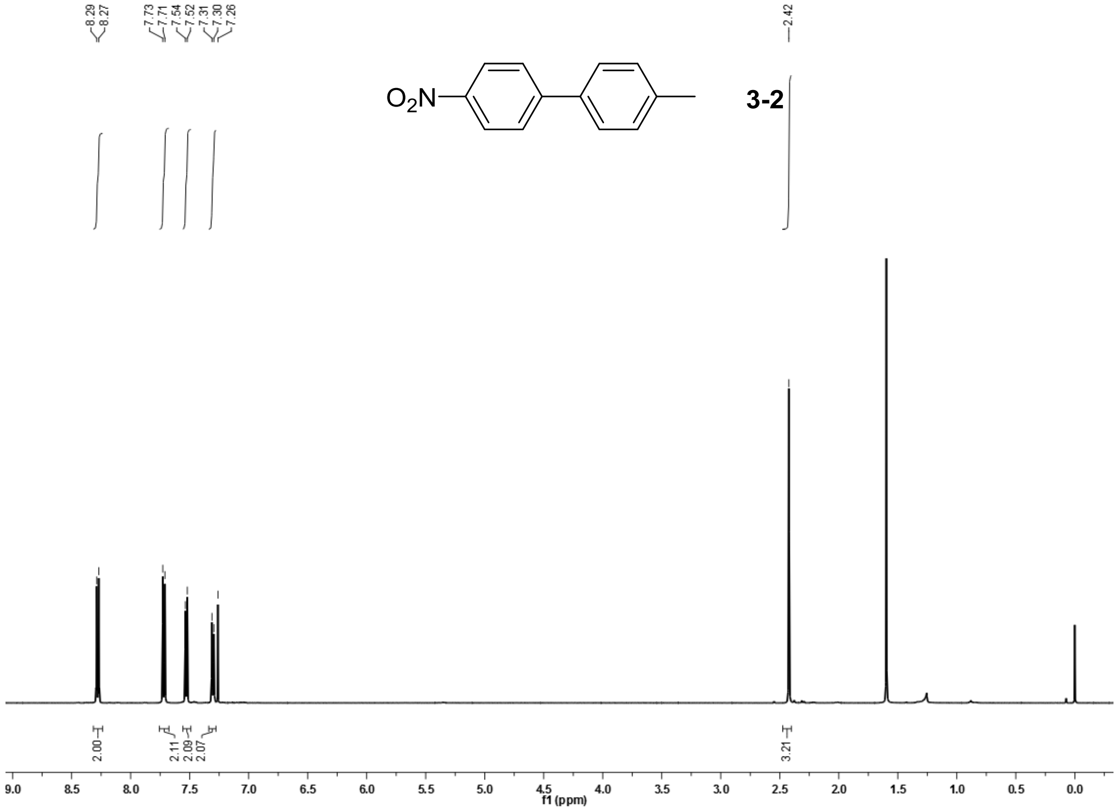

4-Methyl-4'-acetylbiphenyl (**3-3**)[3]

^{1}H NMR ($CDCl_3$, 500 MHz): δ 8.03 (d, *J* = 8.4 Hz, 2H), 7.69 (d, *J* = 8.4 Hz, 2H), 7.55 (d, *J* = 8.0 Hz, 2H), 7.29 (d, *J* = 8.0 Hz, 2H), 2.65 (s, 3H), 2.42 (s, 3H).

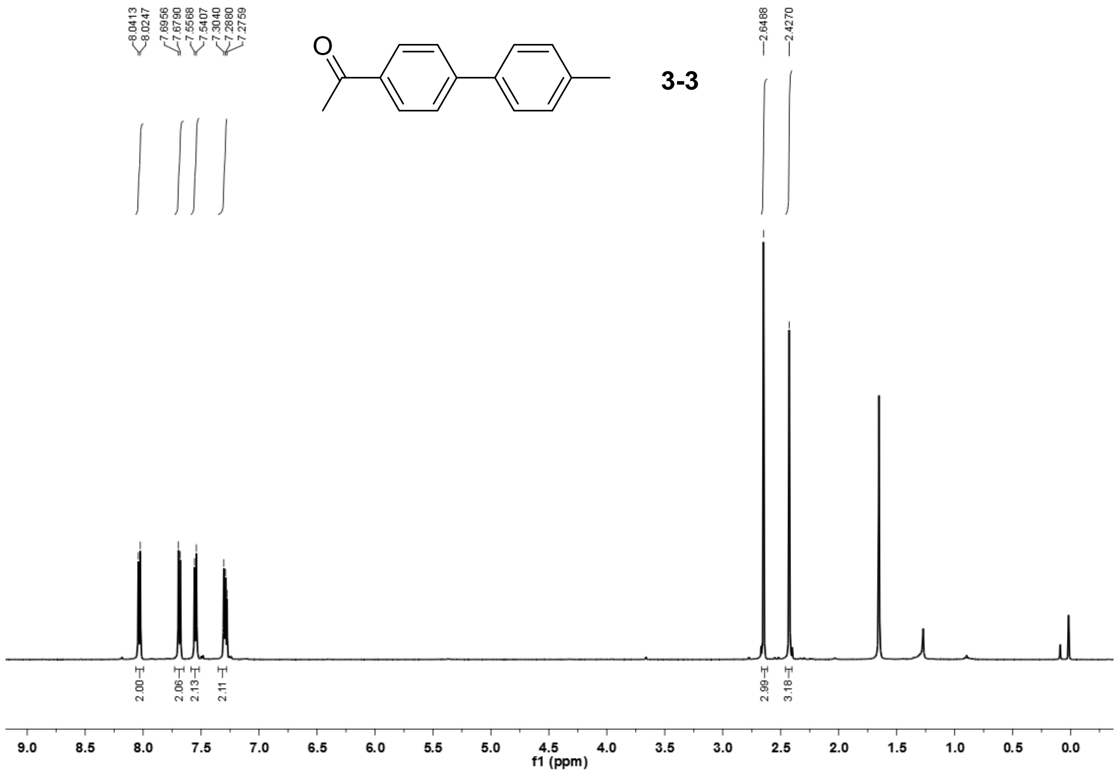

4-Benzoyl-4'-methylbiphenyl (**3d-4**)[4]

^{1}H NMR ($CDCl_3$, 500 MHz): 7.88 (d, *J* = 8.3 Hz, 2H), 7.83 (d, *J* = 7.5 Hz, 2H), 7.69 (d, *J* = 8.3 Hz, 2H), 7.60 (t, *J* = 7.5 Hz, 1H), 7.56 (d, *J* = 8.0 Hz, 2H), 7.50 (t, *J* = 7.5 Hz, 2H), 7.29 (d, *J* = 8.0 Hz, 2H), 2.42 (s, 3H).

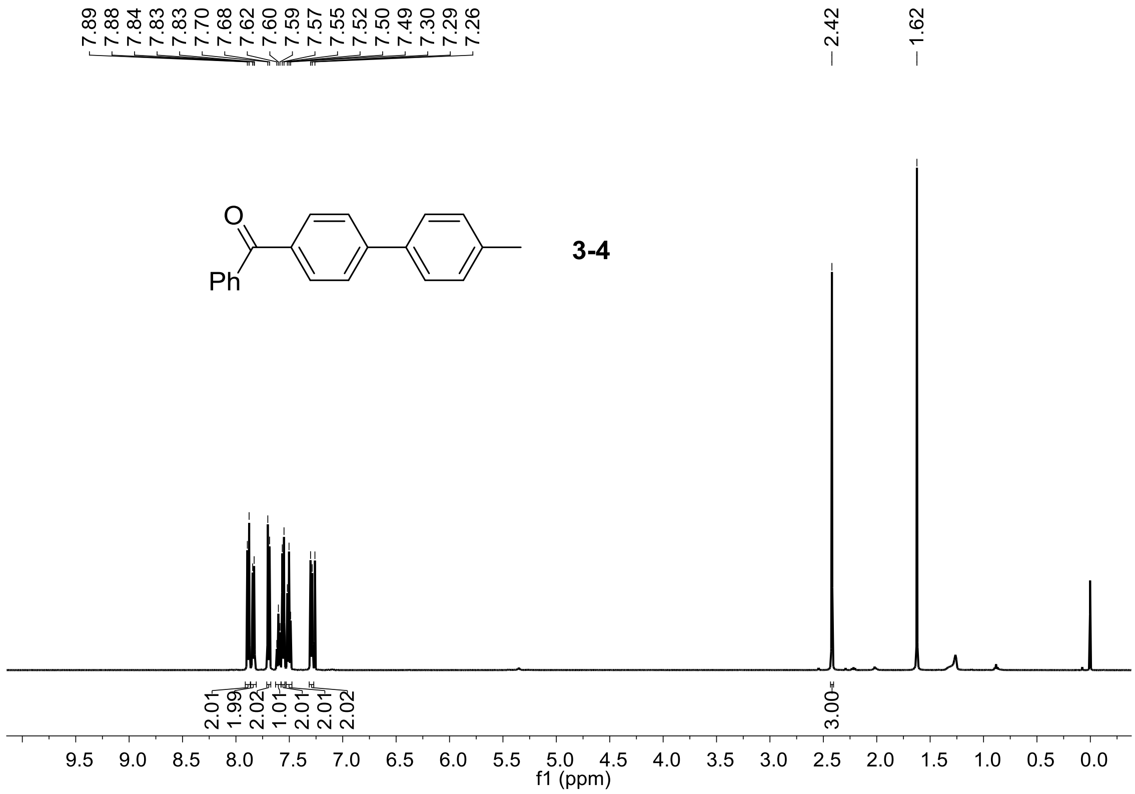

4-Formyl-4'-methylbiphenyl (**3-5**)[5]

^{1}H NMR ($CDCl_3$, 500 MHz): 10.08 (s, 1H), 7.95 (d, *J* = 8.2 Hz, 2H), 7.76 (d, *J* = 8.2 Hz, 2H), 7.56 (d, *J* = 8.1 Hz, 2H), 7.30 (d, *J* = 8.1 Hz, 2H), 2.43 (s, 3H).

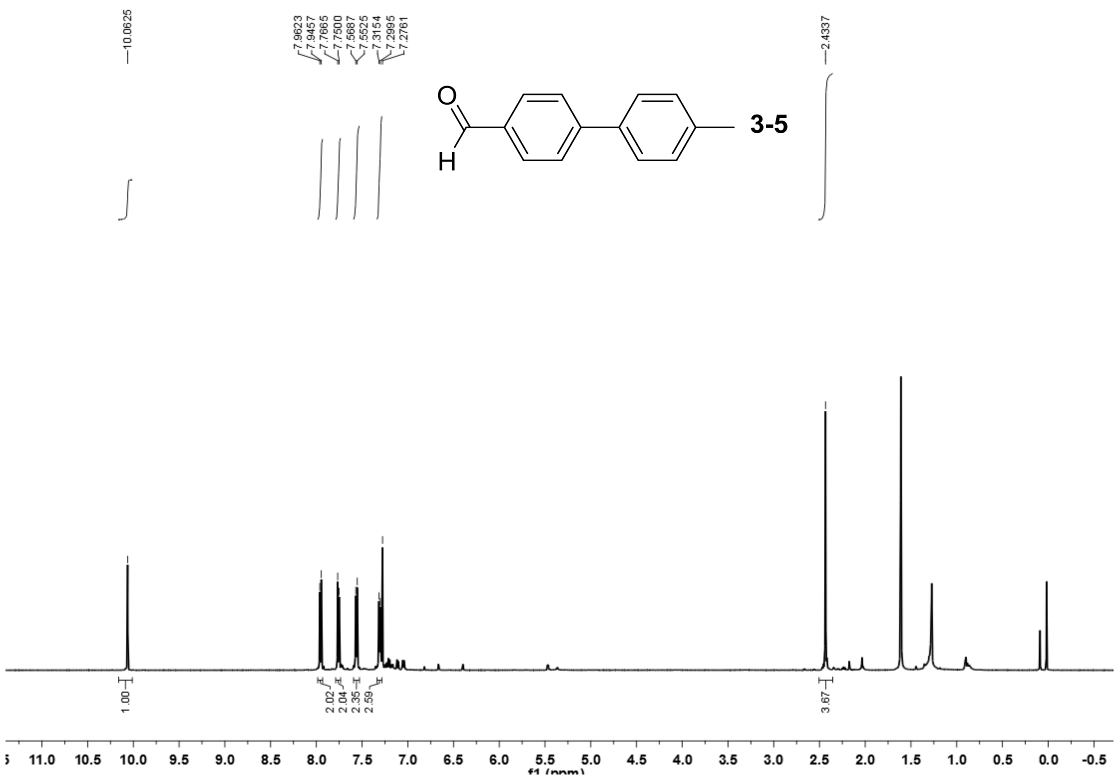

4-Cyano-4'-methylbiphenyl (**3-6**)[4]

^1H NMR ($CDCl_3$, 500 MHz): 7.73-7.64 (m, 4H), 7.49 (d, *J* = 8.0 Hz, 2H), 7.29 (d, *J* = 8.0 Hz, 2H), 2.42 (s, 3H).

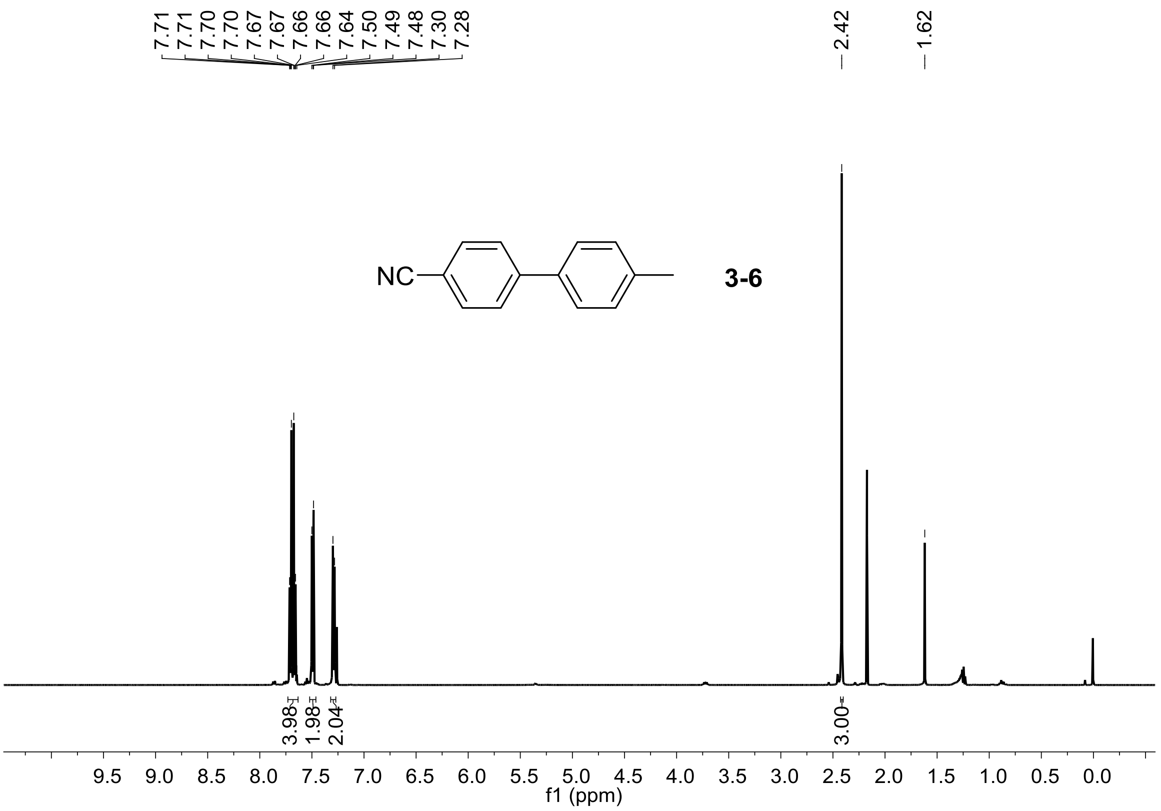

4-Trifluoromethyl-4'-methylbiphenyl (**3-7**)[6]

^{1}H NMR ($CDCl_3$, 500 MHz): 7.69 (br, 4H), 7.51 (d, *J* = 8.1 Hz, 2H), 7.30 (d, *J* = 8.1 Hz, 2H), 2.43 (s, 3H).

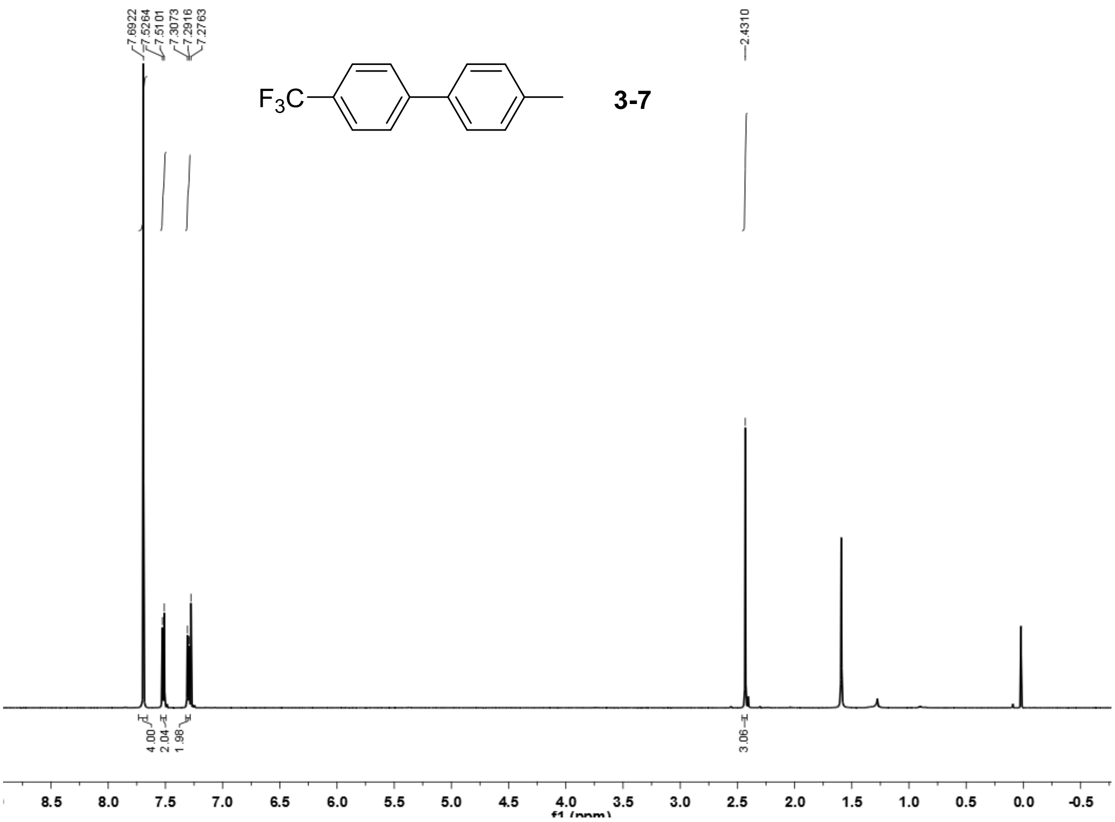

4-Fluoro-4'-methylbiphenyl (**3-8**)[7]

^{1}H NMR ($CDCl_3$, 500 MHz): 7.58-7.51 (m, 2H), 7.45 (d, *J* = 8.1 Hz, 2H), 7.26 (d, *J* = 8.1 Hz, 2H), 7.12 (t, *J* = 8.5 Hz, 2H), 2.41 (s, 3H).

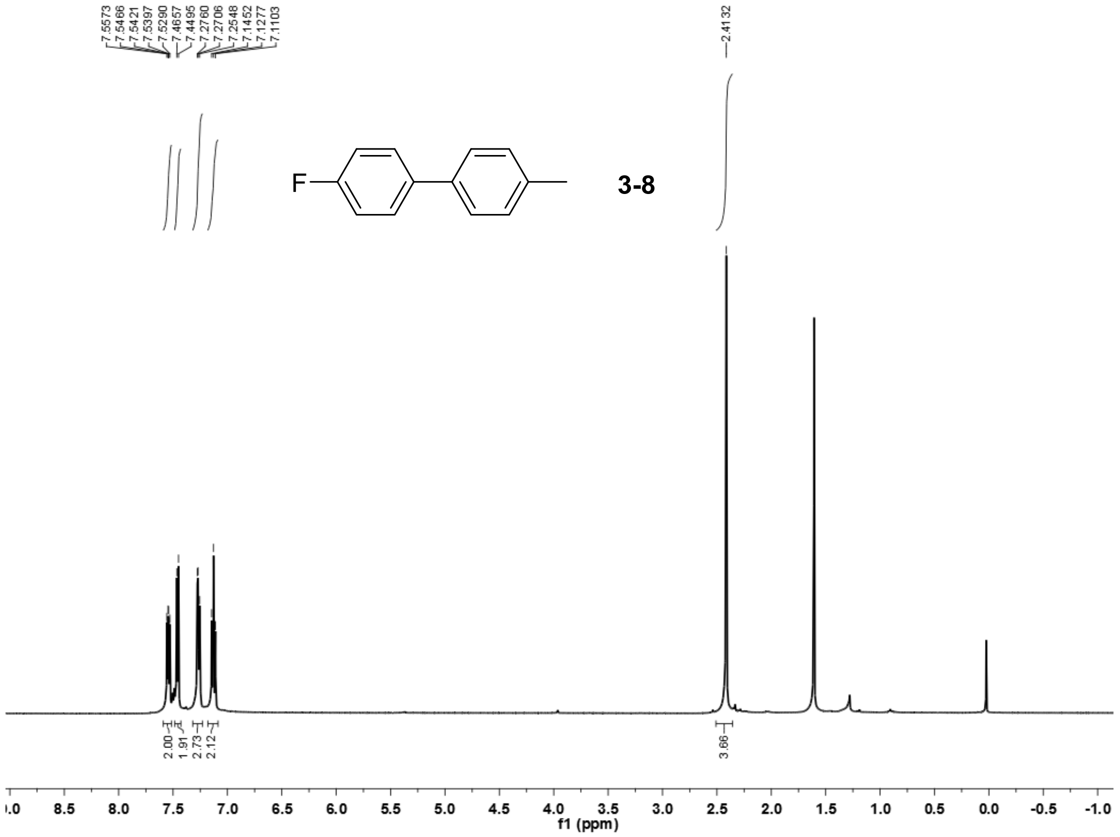

4-Amino-4'-methylbiphenyl (**3-9**)[8]

^{1}H NMR ($CDCl_3$, 500 MHz): 7.45 (d, *J* = 8.1 Hz, 2H), 7.41 (d, *J* = 8.5 Hz, 2H), 7.22 (d, *J* = 8.1 Hz, 2H), 6.77 (d, *J* = 8.5 Hz, 2H), 3.74 (br, 2H), 2.39 (s, 3H).

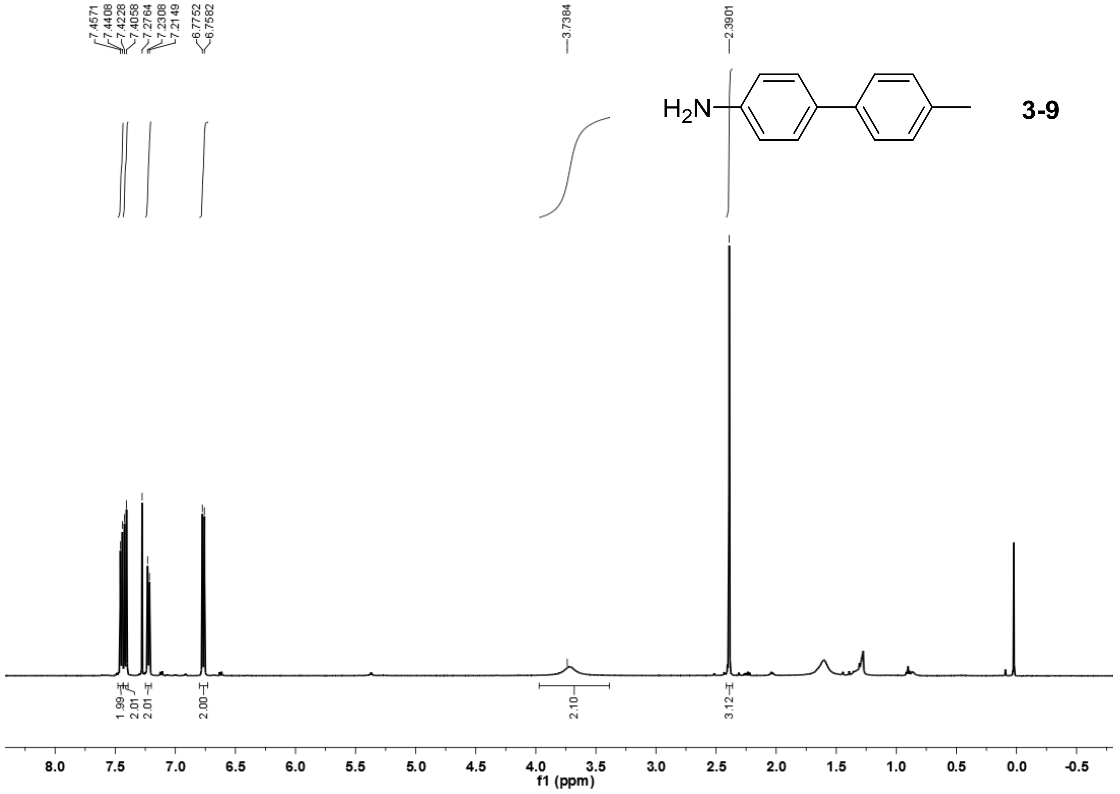

4-Methoxy-4'-methylbiphenyl (**3-10**)[4]

^{1}H NMR ($CDCl_3$, 500 MHz): δ 7.52 (d, *J* = 8.6 Hz, 2H), 7.47 (d, *J* = 8.0 Hz, 2H), 7.24 (d, *J* = 8.0 Hz, 2H), 6.98 (d, *J* = 8.6 Hz, 2H), 3.87 (s, 3H), 2.40 (s, 3H).

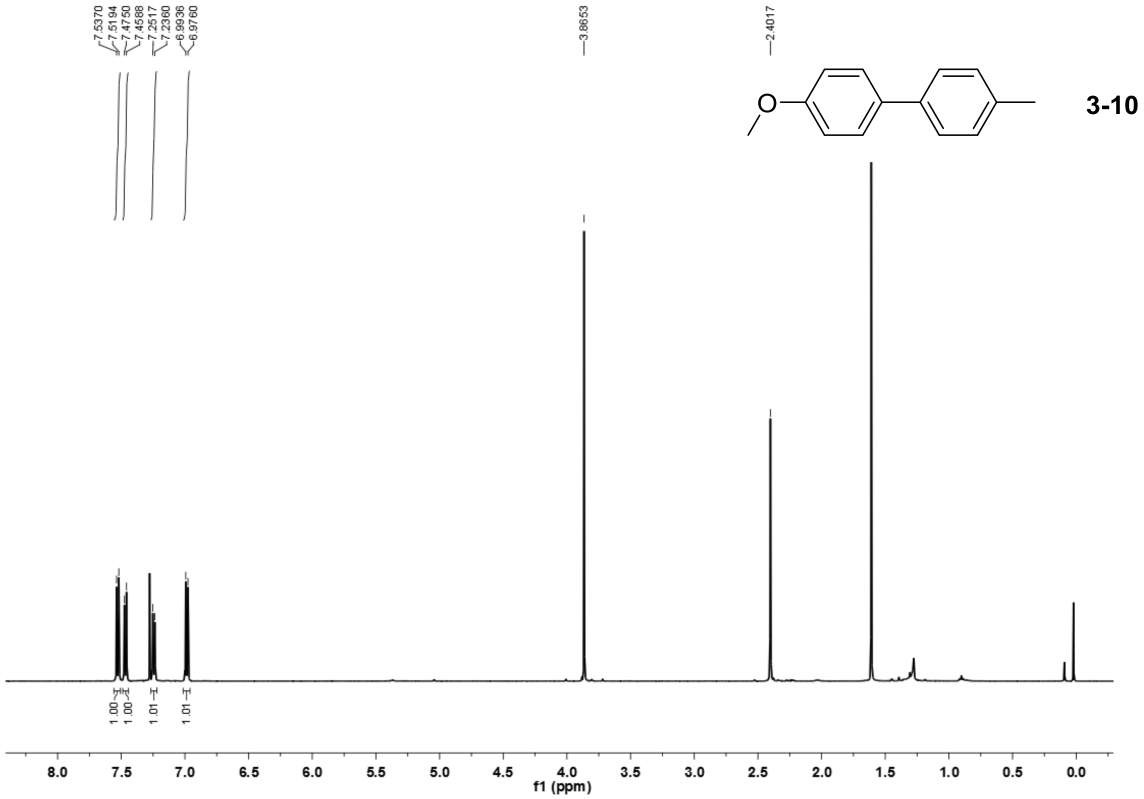

2,4'-Dimethylbiphenyl (**3-11**)[9]

^{1}H NMR ($CDCl_3$, 500 MHz): 7.33-7.21 (m, 8H), 2.43 (s, 3H), 2.31 (s, 3H).

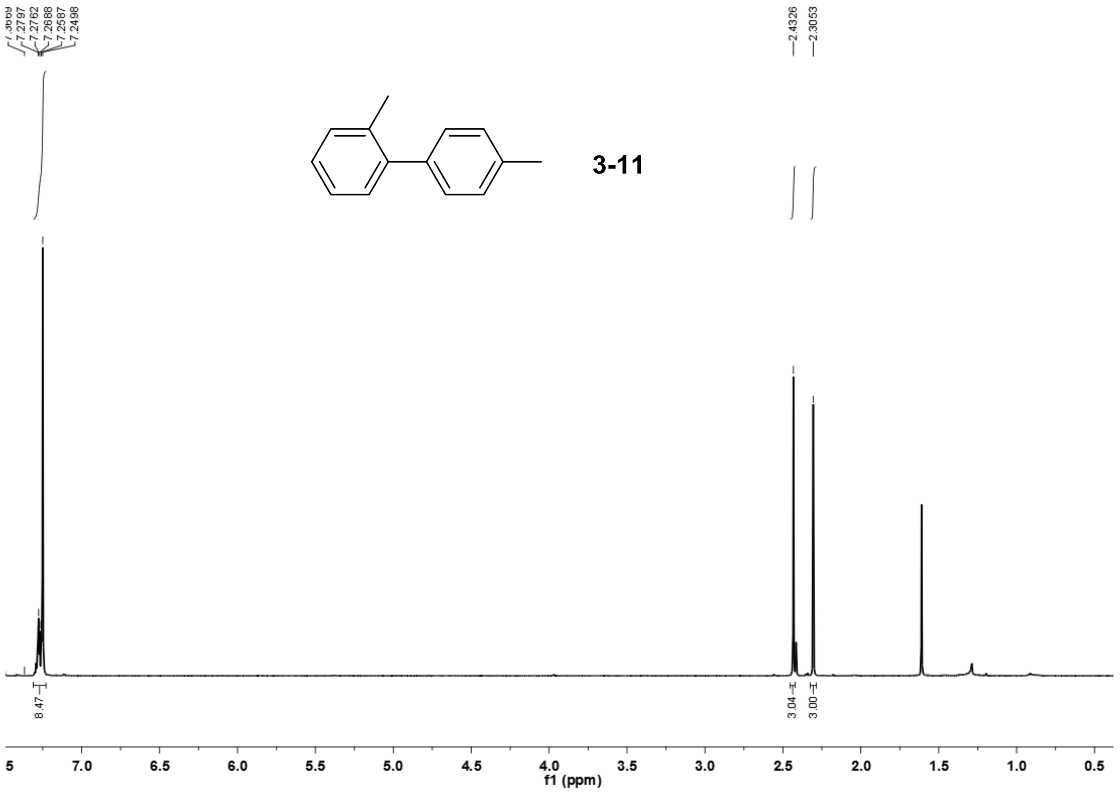

3-Acetyl-4'-methylbiphenyl (**3-12**)[10]

^{1}H NMR ($CDCl_3$, 500 MHz): 8.18 (s, 1H), 7.90 (d, *J* = 7.9 Hz, 1H), 7.78 (d, *J* = 7.9Hz, 1H), 7.56-7.45 (m, 3H), 7.28 (d, *J* = 8.1Hz, 2H), 2.66 (s, 3H), 2.42 (s, 3H).

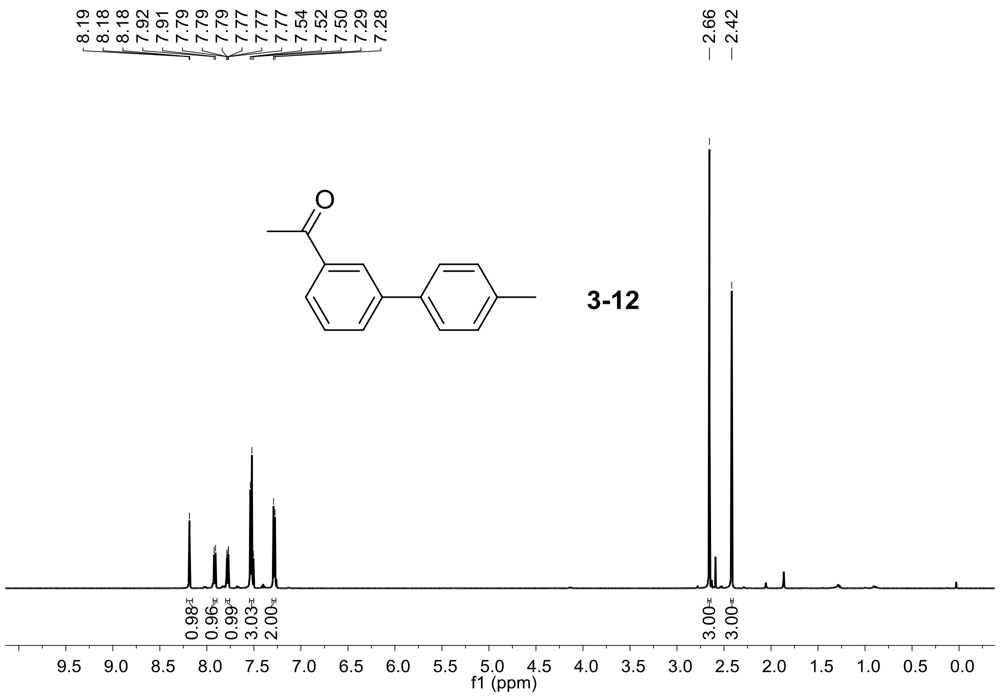

2-(*p*-Tolyl)pyridine (**3-13**)[4]

^{1}H NMR ($CDCl_3$, 500 MHz): 8.69 (d, *J* = 4.7 Hz, 1H), 7.90 (d, *J* = 8.2 Hz, 2H), 7.77-7.69 (m, 2H), 7.29 (d, *J* = 8.2 Hz, 2H), 7.21 (dd, *J*1 = 4.7 Hz, *J*2 = 1.7 Hz, 1H), 2.43 (s, 3H).

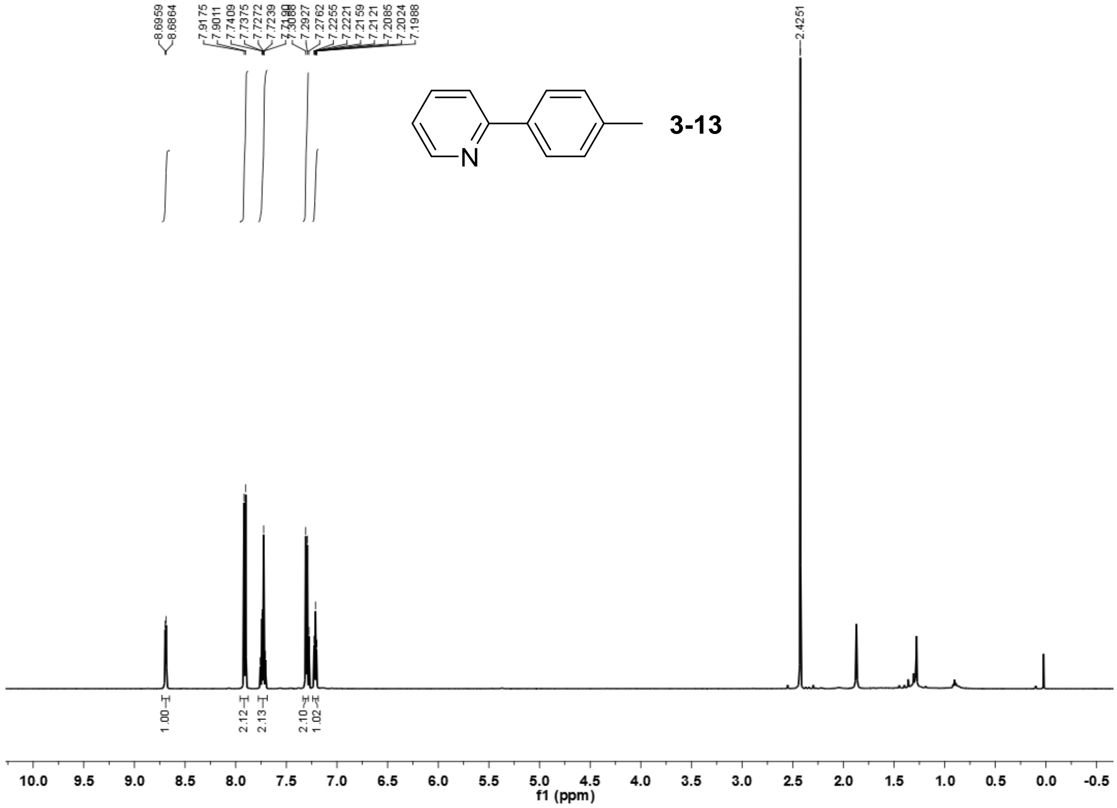

4-Acetyl-4'-trifluoromethylbiphenyl (**3-14**)[11]

^{1}H NMR ($CDCl_3$, 500 MHz): ⍰ 8.08 (d, *J* = 8.5 Hz, 2H), 7.74 (br, 4H), 7.71 (d, *J* = 8.5 Hz, 2H), 2.69 (s, 3H).

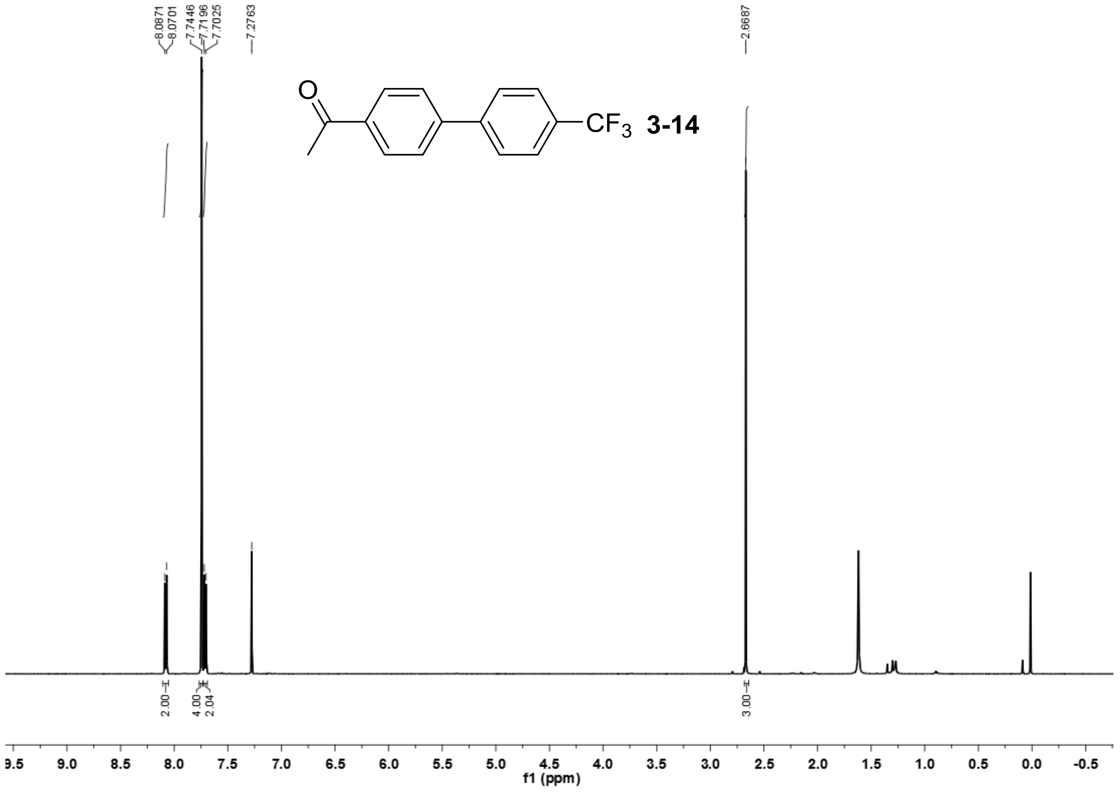

4-Acetyl-4'-(*tert*-butyl)biphenyl (**3-15**)[12]

^{1}H NMR ($CDCl_3$, 500 MHz): ⍰ 8.04 (d, *J* = 8.5 Hz, 2H), 7.70 (d, *J* = 8.5 Hz, 2H), 7.60 (d, *J* = 8.6 Hz, 2H), 7.51 (d, *J* = 8.6 Hz, 2H), 2.65 (s, 3H), 1.40 (s, 9H).

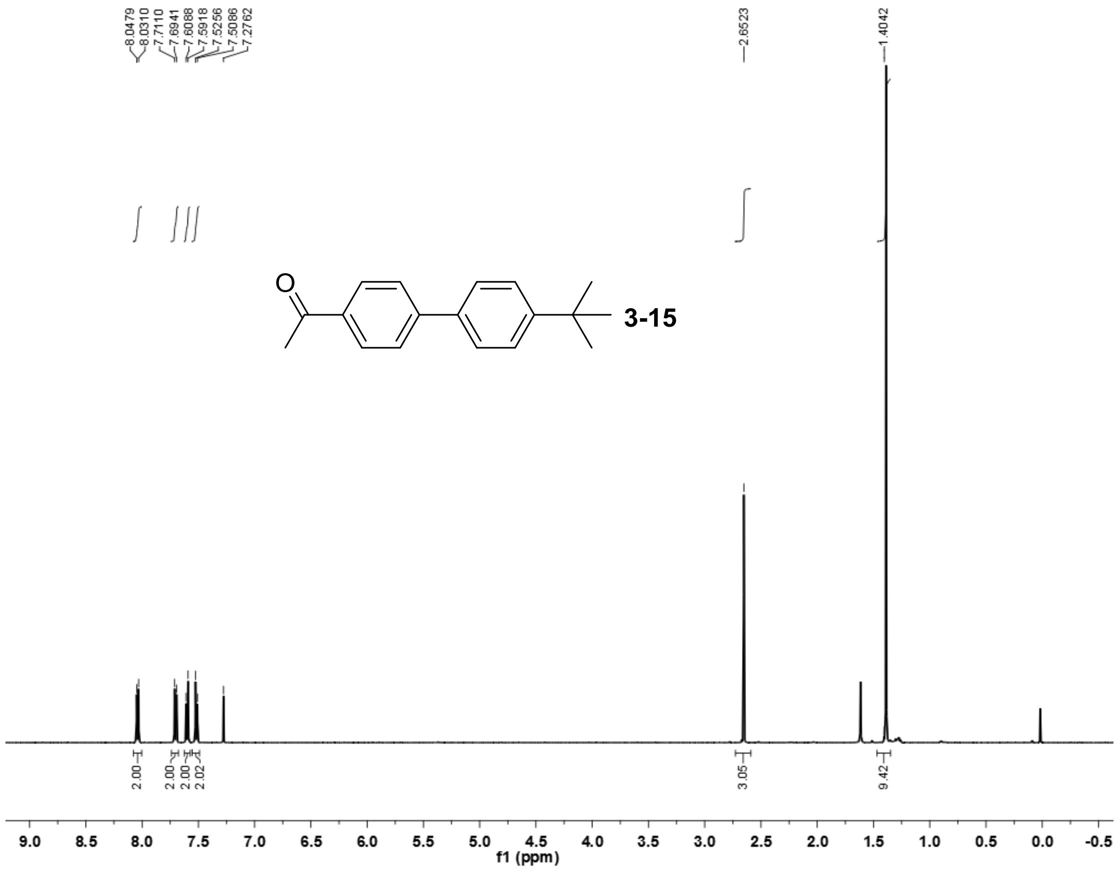

4-Acetyl-4'-methoxylbiphenyl (**3-16**)[10]

^{1}H NMR ($CDCl_3$, 500 MHz): 8.02 (d, *J* = 8.3 Hz, 2H), 7.66 (d, *J* = 8.3 Hz, 2H), 7.59 (d, *J* = 8.8 Hz, 2H), 7.01 (d, *J* = 8.8 Hz, 2H), 3.87 (s, 3H), 2.64 (s, 3H).

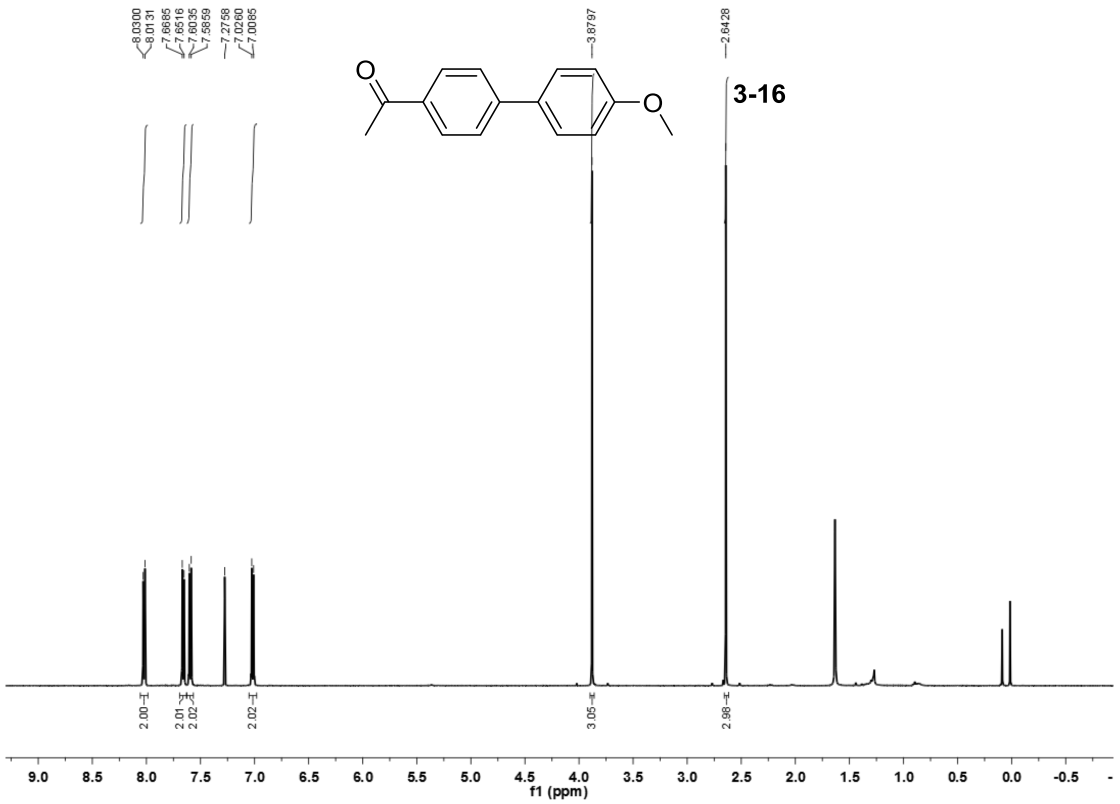

4-Acetyl-2'-methylbiphenyl (**3-17**)[13]

^{1}H NMR ($CDCl_3$, 500 MHz): [illegible] 8.02 (d, *J* = 8.5 Hz, 2H), 7.44 (d, *J* = 8.5 Hz, 2H), 7.32-7.26 (m, 3H), 7.24 (d, *J* = 7.6 Hz, 1H), 2.67 (s, 3H), 2.29 (s, 3H).

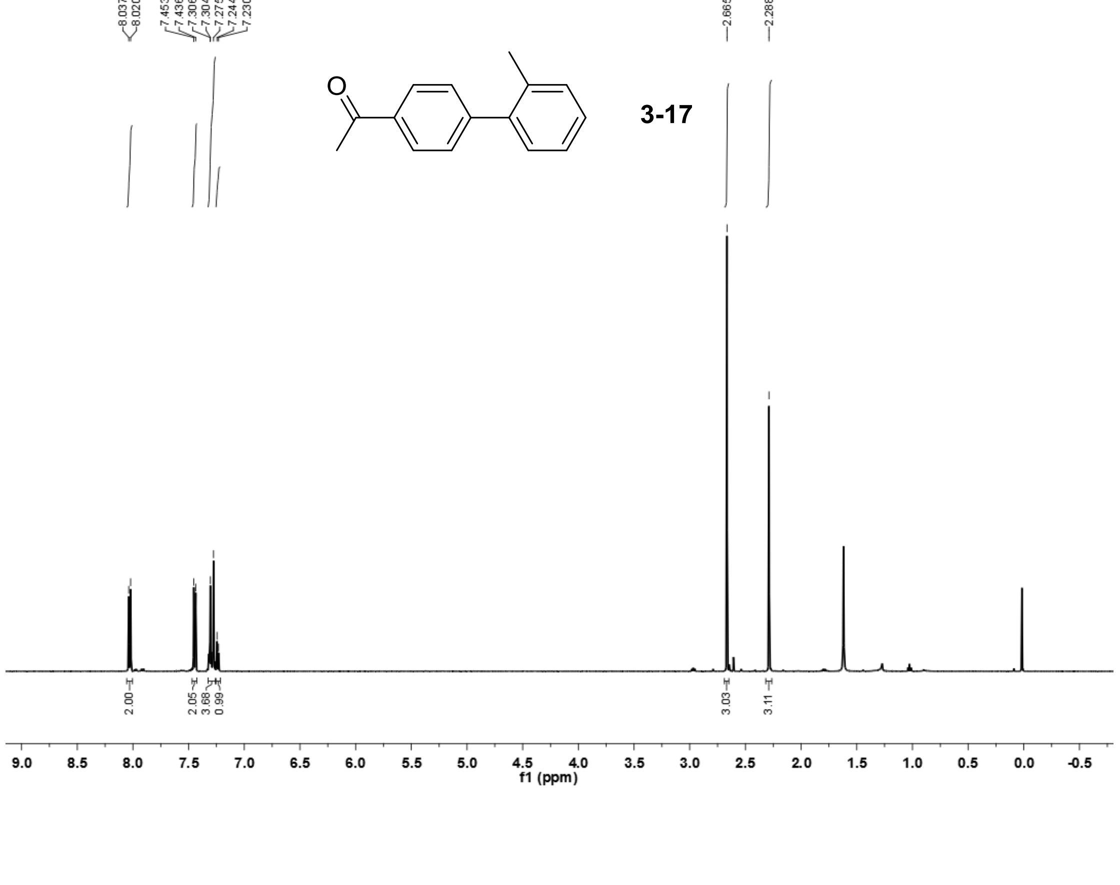

References

1. Y. Tang, Y. Zeng, Q. Hu, F. Huang, L. Jin, W. Mo, N. Sun, B. Hu, Z. Shen, X. Hu and W.-H. Sun, *Adv. Synth. Catal.*, 2016, **358**, 2642-2651.
2. W.-J. Zhou, K.-H. Wang, J.-X. Wang and D.-F. Huang, *Eur. J. Org. Chem.*, 2010, 416-419,
3. G. A. Molander and L. Iannazzo, *J. Org. Chem.*, 2011, **76**, 9182-9187.
4. L.-G. Xie and Z.-X. Wang, *Chem. - Eur. J.*, 2010, **16**, 10332-10336.
5. B. Karimi, D. Elhamifar, J. H. Clark and A. J. Hunt, *Chem. - Eur. J.*, 2010, **16**, 8047-8053.
6. L. Ackermann and A. Althammer, *Org. Lett.*, 2006, **8**, 3457-3460.
7. K. Fuchibe, Y. Ohshima, K. Mitomi and T. Akiyama, *Org. Lett.*, 2007, **9**, 1497-1499.
8. Z. Du, W. Zhou, F. Wang and J.-X. Wang, *Tetrahedron*, 2011, **67**, 4914-4918.
9. C. M. So, C. P. Lau and F. Y. Kwong, *Org. Lett.*, 2007, **9**, 2795-2798.
10. M. L. N. Rao, D. N. Jadhav and D. Banerjee, *Tetrahedron*, 2008, **64**, 5762-5772.
11. S.-N. Chen, W.-Y. Wu and F.-Y. Tsai, *Tetrahedron*, 2008, **64**, 8164-8168.
12. H. Wang, L. Li, X.-F. Bai, W.-H. Deng, Z.-J. Zheng, K.-F. Yang and L.-W. Xu, *Green Chem.* 2013, **15**, 2349-2355.
13. R. Bernini, S. Cacchi, G. Fabrizi, G. Forte, F. Petrucci, A. Prastaro, S. Niembro, A. Shafir and A. Vallribera, *Green Chem.*, 2010, **12**, 150-158.
